# Smart Nanomaterials and Natural Biologics for Innate–Adaptive Immune Reprogramming: A Nanobiotechnology Framework for Translational Medicine

**DOI:** 10.3390/nano16120770

**Published:** 2026-06-18

**Authors:** Kawther Zaher, Mai M. El-Daly, Sherif A. El-Kafrawy, Aymn T. Abbas, Umama A. Abdel-dayem, Zeenat Mirza

**Affiliations:** 1 Immunology Unit, King Fahd Medical Research Center, King Abdulaziz University, Jeddah 21589, Saudi Arabia; 2 Special Infectious Agents Unit-BSL3, King Fahd Medical Research Center, King Abdulaziz University, Jeddah 21589, Saudi Arabia; meldaly@kau.edu.sa (M.M.E.-D.); saelkfrawy@kau.edu.sa (S.A.E.-K.); atabdalhadi@kau.edu.sa (A.T.A.); 3 Animal Facility Unit, King Fahd Medical Research Center, King Abdulaziz University, Jeddah 21589, Saudi Arabia; uabdelsalam@kau.edu.sa; 4 EcoHealth Unit, King Fahd Medical Research Center, King Abdulaziz University, Jeddah 21589, Saudi Arabia; zmirza1@kau.edu.sa; 5 Department of Medical Laboratory Sciences, Faculty of Applied Medical Sciences, King Abdulaziz University, Jeddah 21589, Saudi Arabia

**Keywords:** nanobiotechnology, smart nanomaterials, nanomedicine, innate immunity, adaptive immunity, trained immunity, natural biologics, immunomodulation, dendritic cells, lipid nanoparticles, cancer immunotherapy, translational medicine

## Abstract

The innate–adaptive immune interface is a decisive control point determining whether therapeutic interventions induce durable protection, antitumor immunity, inflammatory, or immune tolerance. Many immunotherapies fail in translation because immunity is often treated as a single-output system rather than a spatially and temporally organized network shaped by tissue context, antigen-presenting cell fate, biomolecular conditioning, and metabolic state. This review introduces the immunoscape framework as a nanobiotechnology-oriented model for linking immune-state mapping with controllable translational variables, including delivery route, release kinetics, first-contact immune cells, lymphatic routing, biomolecular corona identity, antigen-presenting cell fate, and safety-gate assessment. Unlike systems immunology, which primarily describes immune networks, or conventional immune engineering, which often focuses on selected payloads, targets, or platforms, the immunoscape framework provides a design layer for predicting context-dependent immune outcomes. We discuss two converging strategies for reprogramming this interface: natural biologics, including beta-glucans, polyphenols, microbial metabolites, and extracellular vesicles; and smart nanomaterials, including lipid nanoparticles, biomimetic vesicles, lymph node-targeted platforms, and stimulus-responsive nanoarchitectures. We further propose translational design rules to guide clinically realistic immune-reprogramming nanomedicines for cancer, infectious, inflammatory, and regenerative applications.

## 1. Introduction

The classical dichotomy between innate and adaptive immunity, established over a century ago, positioned the innate system as a rapid but non-specific first responder and the adaptive system as a slower but highly specific and memory-endowed defender [1]. This framework, while foundational, has undergone substantial revision in the past two decades. The discovery that innate immune cells, particularly monocytes, macrophages, and natural killer cells, can exhibit enhanced responsiveness upon re-exposure to pathogens challenged the dogma that immunological memory was the exclusive domain of lymphocytes [2,3]. Simultaneously, the recognition that adaptive immune responses are profoundly shaped by the quality and context of innate immune signals has repositioned the innate–adaptive interface as a critical determinant of immune outcomes [4].

Nanomedicine has evolved from largely passive carriers that improve solubility, circulation time, or biodistribution to programmable platforms that actively modulate immune function. This shift is especially relevant because many consequential therapies, vaccines, cancer immunotherapies, gene and RNA medicines, and biologics succeed or fail based on immune interpretation rather than on pharmacokinetics alone. Immune outcomes, however, are rarely determined by a single receptor–ligand pathway. Instead, they emerge from interfaces between innate sensing and adaptive instruction, governed by spatial location (tissue niche), temporal dynamics (kinetics), and cellular sequence (which immune cells encounter signals first). This evolving understanding has coincided with transformative developments in two parallel fields: the characterization of natural biologics as immunomodulatory agents and the engineering of smart nanomaterials capable of precise, spatiotemporal immune modulation [5,6]. Natural biologics, compounds derived from biological sources, including fungal polysaccharides, plant-derived polyphenols, microbe-derived metabolites, and cell-derived extracellular vesicles, have been shown to engage pattern recognition receptors, modulate cytokine networks, and induce durable epigenetic changes in innate immune cells [7,8]. Smart nanomaterials, meanwhile, offer unprecedented control over the delivery, timing, and targeting of immunomodulatory payloads, enabling researchers to reprogram immune cells with precision previously unattainable [9,10].

A central translational bottleneck is the innate–adaptive immune interface: the set of cellular and molecular handoffs that convert early innate detection (pattern recognition, complement activation, inflammasome signaling) into durable adaptive programs (T-cell priming, B-cell maturation, memory formation, tolerance). Two concepts have recently made this interface even more consequential for immunotherapy design. First, trained immunity demonstrates that innate and progenitor compartments can acquire durable memory-like functional states after microbial or metabolic exposures, mediated by coordinated epigenetic and metabolic reprogramming [2,3]. This means that baseline immune responsiveness is not fixed: it can be preconditioned in ways that amplify protective responses or increase inflammatory risk, depending on context, dose, and timing. Second, nanoparticles acquire a dynamic biological identity through the formation of biomolecular coronas in biofluids, which can dominate how materials are opsonized, trafficked, and recognized by immune cells [11,12]. The immune system often responds to the corona rather than the pristine engineered surface, making immune outcomes sensitive to patient-specific plasma composition, disease states, and formulation details. Despite their translational importance, both trained immunity and biomolecular corona formation present major limitations for clinical immune engineering. Trained immunity is not uniformly protective; depending on dose, timing, tissue compartment, and host inflammatory background, it may enhance antimicrobial or antitumor readiness but may also amplify chronic inflammation, myeloid suppression, or immunopathology. Its magnitude and durability can vary substantially among individuals due to age, metabolic status, microbiome composition, prior infection or vaccination history, inflammatory disease status, and baseline myeloid or progenitor-cell conditioning. Similarly, the biomolecular corona introduces a second layer of variability because nanoparticle identity is reshaped by patient-specific plasma proteins, complement components, immunoglobulins, lipoproteins, and disease-associated proteomic changes. As a result, the same engineered nanomaterial may show different cellular uptake, biodistribution, clearance, complement activation, and immune-cell targeting across patients or experimental systems. These challenges make standardized biofluid testing, corona profiling, complement-activation assays, batch-to-batch characterization, and human-relevant immune models essential for improving reproducibility and clinical predictability.

To address these challenges, we propose the concept of immunoscapes: dynamic landscapes of (i) innate sensing pathways, (ii) biomolecular conditioning (including corona formation), (iii) cellular trafficking and tissue retention, (iv) antigen-presenting cell (APC) fate decisions, and (v) immunometabolic constraints that collectively shape adaptive outcomes [13]. The immunoscape concept differs from existing systems immunology and immune-engineering paradigms in its intended use. Systems immunology provides high-dimensional descriptions of immune networks, cellular states, and signaling interactions, whereas immune engineering usually focuses on manipulating selected immune pathways, cells, antigens, adjuvants, or delivery systems. The immunoscape framework bridges these approaches by translating immune-network information into design rules for nanobiotechnology. In this framework, the therapeutic outcome is treated as the product of coordinated variables: where the material travels, how long the signal persists, which immune cell interprets it first, how the biomolecular corona modifies its biological identity, how APCs are conditioned, and whether the resulting adaptive program is protective, tolerogenic, exhausted, inflammatory, or antitumor. The translational value of this framework is supported by several convergent observations discussed in this review: lymph-node-targeted nanoparticles can alter antigen persistence and adaptive immune quality; LNPs can act as both delivery vehicles and innate immune activators; biomolecular corona formation can redirect immune recognition and clearance; and trained-immunity stimuli can durably reset innate responsiveness. Together, these examples show that immune-reprogramming outcomes are governed by spatial, temporal, cellular, and material-context variables, not by payload identity alone.

The immunoscape framework is intentionally translational: it encourages designers to treat immune programming as a controllable system defined by space (where), time (when), and sequence (who encounters what first), rather than as a single ‘immunostimulatory versus immunosuppressive’ dial. At its current stage, the immunoscape framework should be viewed as an integrative translational model rather than a fully validated standalone experimental system. Its individual components are supported by substantial experimental and clinical evidence: trained-immunity studies show that innate immune setpoints can be durably reshaped; lymph-node-targeted nanoparticles demonstrate that spatial routing and antigen persistence influence adaptive immune quality; lipid nanoparticle systems show that delivery platforms can function as both carriers and innate immune modulators; biomolecular corona studies demonstrate that patient- and matrix-dependent surface conditioning can redirect immune recognition; and dendritic-cell and macrophage reprogramming studies confirm that early myeloid interpretation can determine downstream T-cell and humoral outcomes. However, these mechanisms have not yet been prospectively integrated and tested as a unified immunoscape-guided design pipeline. Therefore, the framework is presented here as a synthesis that translates validated biological and material principles into practical design rules. Future studies should determine whether immunoscape-guided formulation improves prediction of immune phenotype, biodistribution, safety, and therapeutic efficacy compared with conventional cargo- or platform-centered development.

Because adaptive immunity is largely orchestrated in secondary lymphoid organs, lymphatic transport is a strategic lever for immunoscape engineering [14]. Lymphatic vessels continuously transport antigens, particulates, and immune cells from peripheral tissues to draining lymph nodes, where APCs coordinate T- and B-cell responses. The efficiency of lymphatic entry and lymph node accumulation is strongly influenced by the physicochemical properties of nanoparticles, particularly size, surface characteristics, and formulation-dependent interactions with interstitial matrices and the lymphatic endothelium [14,15]. A recurring design principle in lymphatic delivery is that nanoparticle size strongly influences interstitial transport, lymphatic entry, and lymph-node retention, although the optimal range is platform- and environment-dependent. Very small particles, typically below approximately 5–10 nm, may diffuse rapidly into blood capillaries or pass through lymph nodes with limited retention. By contrast, nanoparticles in the approximate 10–100 nm range often show more efficient lymphatic drainage and lymph-node access after interstitial administration, with particles around 20–50 nm frequently considered favorable for lymphatic entry, nodal penetration, and interaction with resident antigen-presenting cells. Larger particles, particularly those above approximately 100–200 nm, may undergo slower transport through the extracellular matrix, show greater retention at the injection site, or be preferentially captured by local phagocytes. These ranges should not be interpreted as fixed thresholds. Lymphatic routing can shift according to material composition, surface charge, PEGylation, biomolecular corona formation, shape, rigidity, polydispersity, injection route, tissue matrix density, inflammatory status, lymphatic permeability, and disease-associated remodeling. Therefore, size optimization should be performed for each nanomaterial platform and intended biological environment rather than applied as a universal rule. This size-linked routing matters because it affects not only “how much reaches the lymph node,” but also which APC subsets encounter the material first, how long antigen persists, and whether the immune system experiences a short pulse versus sustained presentation. In vaccine contexts, lymph node-targeting nanoparticle systems can enhance antigen persistence and promote humoral immunity, illustrating how delivery design directly tunes adaptive immune responses.

The clinical success of mRNA–lipid nanoparticle systems has catalyzed a revolution in immune engineering, demonstrating that synthetic nanomaterials can reprogram cellular function with remarkable precision and scalability [16,17]. The rapid expansion of this technology also revealed a critical principle: lipid nanoparticles are not immunologically inert. Mechanistically, LNP-mediated innate immune activation can arise from both the delivery platform and the nucleic acid cargo. Ionizable lipids can perturb endosomal membranes during endosomal escape, promoting cellular stress responses and activation of innate inflammatory pathways. Depending on lipid chemistry, dose, route, and target tissue, LNPs may induce type I interferon signaling, pro-inflammatory cytokine release, complement activation, inflammasome-associated responses, and recruitment of monocytes and other innate immune cells. The RNA cargo can also contribute to innate sensing through endosomal and cytosolic RNA-recognition pathways, particularly when nucleotide modification, RNA purity, double-stranded RNA contaminants, or intracellular localization are not optimally controlled. Excipients, including PEG-lipids and helper lipids, may further influence protein adsorption, complement activation, anti-PEG antibody formation, biodistribution, and clearance. These mechanisms are not necessarily undesirable; in prophylactic or therapeutic vaccination, controlled innate activation can provide adjuvant activity and support adaptive priming. In contrast, for repeated administration, chronic inflammatory diseases, protein-replacement strategies, gene editing, or tolerogenic applications, the same immune activation may become a liability by increasing reactogenicity, accelerating clearance, reducing repeated-dose expression, altering biodistribution, or increasing cumulative inflammatory risk. They can stimulate innate pathways that influence adaptive outcomes, sometimes beneficially (in vaccination contexts) and sometimes problematically (in repeated dosing, inflammatory liabilities, or chronic indications) [18]. For translational reviews, it is therefore insufficient to discuss nanocarriers in general terms. The platform’s intrinsic immune activity of the platform, the contribution of excipients (including PEG-lipids), and the interaction between platform immunogenicity and payload biology must be treated as first-order design constraints. Immune outcomes are best understood as immunoscapes, spatiotemporally organized systems where innate sensing, biomolecular conditioning, trafficking, APC fate, and immunometabolism collectively determine adaptive quality. Therefore, LNP design should be stratified by clinical intent: immunostimulatory formulations may be appropriate for vaccines and cancer immunotherapy, whereas repeat-dose therapeutics require minimized innate activation, careful excipient selection, complement testing, anti-PEG or anti-lipid antibody monitoring, and assessment of whether prior exposure changes subsequent pharmacokinetics, biodistribution, and immune safety.

**Figure 1:**
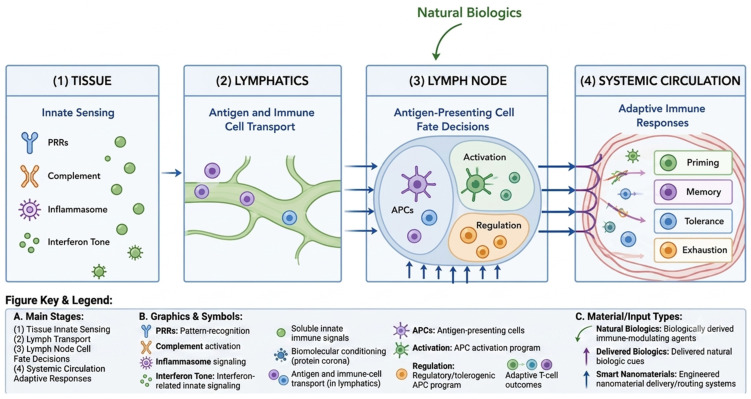
Immunoscape map of innate–adaptive interface control nodes. Immunoscapes are dynamic landscapes where innate sensing, biomolecular conditioning (corona), trafficking, APC fate, and immunometabolism collectively determine adaptive outcomes. Natural biologics tune immune setpoints and resolution programs; smart nanomaterials impose spatiotemporal control through routing, targeting, and release kinetics.

The convergence of natural biologics and smart nanomaterials represents a paradigm in nanobiotechnology for translational medicine. Rather than treating innate and adaptive immunity as separate therapeutic targets, emerging strategies seek to reprogram the interface between these compartments by combining biologically derived immune cues with engineered control over biodistribution, cellular targeting, release kinetics, and safety [19,20]. This review provides a comprehensive examination of this frontier, integrating immunology, materials science, and translational medicine to map the immunoscapes that define the future of immune modulation. Figure 1 presents the immunoscape map and highlights the main control nodes linking innate sensing, trafficking, APC decisions, and adaptive outcomes.

## 2. The Innate–Adaptive Immune Interface and Trained Immunity as a Reprogrammable Control System

### 2.1. Architecture and Communication Across the Innate–Adaptive Interface

The innate–adaptive immune interface is not a single anatomical or molecular boundary but rather a distributed network of cellular interactions, soluble mediators, and tissue-specific microenvironments that collectively determine the magnitude, quality, and duration of immune responses [1,4]. At the cellular level, this interface is anchored by professional antigen-presenting cells, principally dendritic cells (DCs) and macrophages, which serve as sentinels of the innate immune system while simultaneously instructing the adaptive immune compartment through antigen presentation and co-stimulatory signaling [21]. The immune interface is dominated by APCs and dendritic cells that convert early innate cues into adaptive fate decisions.

In practice, immune programming can be understood through four controllable axes that define the immunoscape. First, signal composition encompasses pattern-recognition receptor signaling, complement engagement, inflammasome/interleukin-1 family activity, and interferon tone. Innate activation is not binary; different patterns of PRR activation, complement engagement, and inflammasome signaling encode qualitatively different APC programs [3,5,21,22]. In nanomedicine design, composition includes both the intended cargo (adjuvant, RNA, metabolite, phytochemical) and unintended immune cues (platform immunogenicity, impurities, corona composition) [4,9,23,24]. Second, signal kinetics refers to transient pulses versus sustained stimulation, which often distinguish priming from tolerance and exhaustion. Duration and cadence of stimulation frequently separate productive priming from tolerance or dysfunction: sustained innate stimulation can drive chronic inflammation, suppressive myeloid programs, or T-cell exhaustion, whereas appropriately timed pulses can gate APC maturation and enable durable memory [2,25,26].

Third, signal geography determines whether cues reach draining lymph nodes or remain trapped in peripheral tissue, directly influencing adaptive quality, since lymph nodes are the primary sites of naïve T- and B-cell priming and affinity maturation [1,19,27,28]. A translational consequence is that the same biologic can yield divergent outcomes when delivered systemically versus mucosally, as a pulse versus a depot, or with lymph node access versus peripheral sequestration. Fourth, cellular sequence, the critical principle of “who sees what first”, determines which compartments sense the intervention initially. If particles are captured predominantly by tissue macrophages, antigen may be sequestered or presented in a non-priming context; if migratory DCs or specific nodal DC subsets dominate early uptake, cross-presentation and priming may improve [27,29,30].

Dendritic cells occupy a particularly strategic position at this interface. Upon encountering pathogen-associated molecular patterns (PAMPs) or damage-associated molecular patterns (DAMPs), DCs undergo maturation, upregulating major histocompatibility complex (MHC) molecules and co-stimulatory receptors, including CD80, CD86, and CD40 [21,22]. This maturation process converts DCs from antigen-capturing sentinels into potent T cell activators, effectively translating innate danger signals into adaptive immune programs. The cytokine milieu produced by DCs during this transition, including interleukin-12 (IL-12), IL-6, and type I interferons, further shapes the polarization of T helper cell (Th) responses, determining whether immunity skews toward Th1, Th2, Th17, or regulatory T cell phenotypes [4,22].

The soluble mediators orchestrating communication across this interface include not only classical cytokines and chemokines but also metabolites, complement components, and extracellular vesicles [31,32]. Recent work has highlighted the importance of metabolic crosstalk, in which metabolites such as lactate, succinate, and itaconate produced by activated innate cells can directly influence T cell differentiation and effector functions [33]. Similarly, complement fragments generated during innate immune activation serve as co-stimulatory signals for T and B cells, linking the ancient complement cascade to modern adaptive responses [1].

The tissue microenvironment adds another layer of complexity to the innate–adaptive interface. In the tumor microenvironment (TME), immunosuppressive signals from tumor cells, regulatory T cells, and myeloid-derived suppressor cells (MDSCs) can subvert normal crosstalk between innate and adaptive immunity, creating a state of immune tolerance that facilitates tumor escape [34,35]. Understanding and therapeutically modulating these tissue-specific interfaces has become a central goal of contemporary immunology and a driving motivation for the development of smart nanomaterial-based interventions [9,10].

### 2.2. Trained Immunity: Epigenetic and Metabolic Reprogramming of Innate Cells

The concept of trained immunity, first formally articulated by Netea and colleagues, describes the capacity of innate immune cells to develop enhanced functional responses following initial stimulation, mediated not by genetic recombination but by epigenetic modifications and metabolic rewiring [2,3]. This discovery fundamentally expanded the conceptual framework of immunological memory, demonstrating that the innate system possesses a form of functional adaptation that, while distinct from the clonal memory of T and B cells, can confer durable, broad-spectrum protection against secondary challenges [36].

Trained immunity is one of the clearest examples of durable setpoint shifting in innate compartments [3,5]. It demonstrates that baseline immune responsiveness is not fixed but can be preconditioned in ways that amplify protective responses or increase inflammatory risk, depending on context, dose, and timing [3,5]. The molecular mechanisms of trained immunity have been extensively characterized using the β-glucan model. When monocytes are exposed to β-glucan, a cell wall component of *Candida albicans* recognized by the C-type lectin receptor Dectin-1, a signaling cascade is initiated through the Akt/mTOR/HIF-1α pathway [7,37]. This signaling axis drives a fundamental metabolic shift from oxidative phosphorylation to aerobic glycolysis (the Warburg effect) and simultaneously activates the mevalonate pathway, generating metabolic intermediates that serve as substrates for epigenetic enzymes [36,38]. Although the Akt/mTOR/HIF-1α axis is central to the metabolic shift that supports β-glucan-induced trained immunity, it operates within a broader signaling and epigenetic network. Upstream Dectin-1 engagement activates Syk and CARD9-dependent pathways, leading to activation of NF-κB and MAPK and the induction of inflammatory transcriptional programs. IL-1β signaling can further amplify trained responses and support myeloid progenitor reprogramming, while inflammasome-related pathways may influence the intensity and durability of innate immune training. The mevalonate pathway also contributes to trained immunity by linking intermediates of cholesterol biosynthesis to epigenetic and inflammatory remodeling. At the chromatin level, trained immunity is associated with increased accessibility at promoters and enhancers of inflammatory and antimicrobial genes, enrichment of activating histone marks such as H3K4me3 and H3K27ac, enhancer remodeling, and altered activity of metabolite-sensitive epigenetic enzymes, including histone methyltransferases, demethylases, and acetyltransferases. These mechanisms are therapeutically significant because they provide potential intervention points to enhance vaccine responsiveness, improve antimicrobial or antitumor readiness, reverse endotoxin tolerance or immune paralysis, and reprogram suppressive myeloid compartments. However, the same pathways require careful control because excessive or persistent activation may contribute to chronic inflammation, tissue injury, or immunopathology.

**Figure 2:**
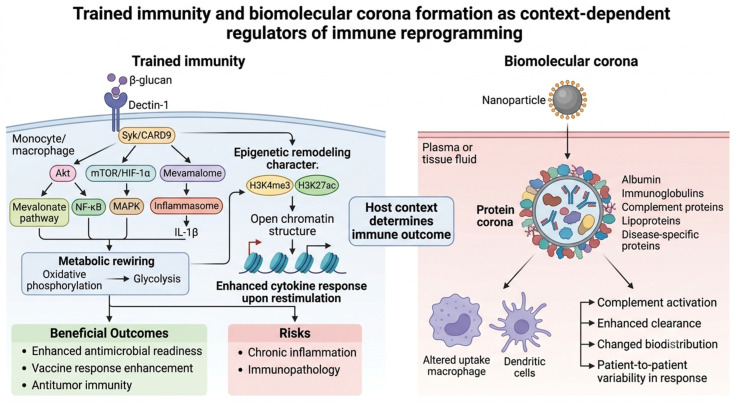
Trained immunity and biomolecular corona formation as context-dependent regulators of immune reprogramming. β-glucan and related immune-training cues activate Dectin-1/Syk/CARD9, Akt/mTOR/HIF-1α, NF-κB, MAPK, inflammasome-linked pathways, IL-1β amplification, and mevalonate-pathway remodeling. These signals reshape chromatin accessibility and epigenetic marks such as H3K4me3 and H3K27ac, producing trained innate states that may enhance antimicrobial, vaccine, or antitumor immunity but may also amplify chronic inflammation if uncontrolled. In parallel, nanoparticles acquire biomolecular coronas from patient-specific biofluids, altering immune recognition, complement activation, biodistribution, cellular uptake, and clearance. Together, trained immunity and corona formation illustrate how immune outcomes are shaped by host context, material identity, and reproducibility constraints.

The context-dependent nature of trained immunity and biomolecular corona formation is summarized in Figure 2. The figure illustrates how β-glucan-driven signaling and epigenetic remodeling can generate trained innate immune states, while nanoparticle corona formation can reshape biological identity, immune recognition, complement activation, cellular uptake, biodistribution, and clearance. Together, these mechanisms demonstrate that immune-reprogramming outcomes depend not only on the intended biologic or nanomaterial payload but also on host inflammatory background, patient-specific biofluid composition, material surface conditioning, and reproducibility constraints.

Specifically, fumarate accumulation inhibits the histone demethylase KDM5, leading to sustained enrichment of the activating histone mark H3K4me3 at the promoters of pro-inflammatory genes such as TNF and IL-6 [7,38]. Concurrently, acetyl-CoA produced through enhanced glycolysis fuels histone acetyltransferase activity, promoting the deposition of H3K27ac at enhancer regions [36]. Thus, trained immunity is best understood not as a single-pathway phenomenon but as an integrated metabolic–epigenetic–transcriptional state that can be therapeutically exploited only when dose, timing, cellular compartment, and inflammatory background are carefully controlled. These epigenetic modifications are remarkably stable, persisting for weeks to months and enabling trained monocytes and macrophages to mount heightened cytokine responses upon re-stimulation with unrelated pathogens [2,3,36]. Importantly, trained immunity is not restricted to peripheral blood monocytes. Studies in murine models have demonstrated that systemic administration of β-glucan reprograms myeloid progenitors in the bone marrow, expanding myelopoiesis through elevated IL-1β and granulocyte-macrophage colony-stimulating factor (GM-CSF) signaling [27]. This central training ensures that the progeny of reprogrammed progenitors inherit the trained phenotype, providing a sustained source of functionally enhanced innate effectors [3,27].

The therapeutic implications of trained immunity are profound. In the context of infectious disease, *Bacillus Calmette–Guérin* (BCG) vaccination, the prototypical trained immunity inducer, has been shown to confer heterologous protection against non-mycobacterial infections, reduce all-cause neonatal mortality, and enhance responses to subsequent vaccinations [2,28]. BCG-induced trained immunity has been reported in humans and is often discussed in the context of heterologous immune effects, offering clinical validation of durable baseline shifts [39,40]. The key translational lesson is not that BCG is a universal strategy, but that durable baseline shifts can be clinically meaningful while remaining population- and context-dependent [39,40]. This variability motivates the development of engineered systems that reproduce beneficial state changes with tighter control.

In oncology, trained immunity has been explored as a strategy to overcome the immunosuppressive TME. Recent work demonstrated that glucan-induced epigenetic and metabolic rewiring of macrophages enhanced colorectal cancer vaccine responses by promoting pro-inflammatory repolarization and augmenting antigen presentation [41]. These findings suggest that trained immunity can serve as a bridge between innate activation and adaptive immune potentiation, effectively reprogramming the innate–adaptive interface to favor adaptive immune responses [3,36]. A trained-innate state can be beneficial if it enhances the competence of antigen-presenting cells and supports immunogenic myeloid programs. Still, it can also be detrimental if it amplifies chronic inflammatory suppression. The core design challenge is therefore controlled dose, timing, and compartment targeting [3,5].

## 3. Natural Biologics as Immune Setpoint Modulators

Natural biologics frequently act as immune setpoint modulators rather than classic single-target drugs: they reshape baseline inflammatory tone, resolution capacity, APC conditioning, and helper/effector polarization. In cancer immunotherapy, this is highly relevant because baseline myeloid bias and tissue inflammation often determine whether checkpoint blockade, vaccination, or adoptive strategies are effective [2,29]. The immunoscape framework emphasizes that natural biologics tune immune setpoints and resolution programs while smart nanomaterials act as context controllers.

### 3.1. β-Glucans and Fungal Polysaccharides

Among natural biologics, β-glucans occupy a preeminent position as immunomodulators capable of reprogramming innate immune function. These glucose polymers, found in the cell walls of fungi, bacteria, and cereals, are recognized by a suite of pattern recognition receptors, including Dectin-1, complement receptor 3 (CR3), and toll-like receptors (TLRs) [7,37]. Engagement of Dectin-1 by particulate β-glucan triggers the Syk–CARD9 signaling axis, leading to NF-κB activation and the production of pro-inflammatory cytokines, while also initiating the metabolic and epigenetic reprogramming characteristic of trained immunity [3,37].

Recent investigations have refined the understanding of how β-glucan structure influences immunological outcomes. Saccharomyces cerevisiae-derived β-glucans have been shown to induce potent trained immunity characterized by enhanced TNF and IL-6 production upon secondary stimulation, with the magnitude of the response correlating with the degree of β-1,3-linked branching [3]. Polysaccharide adjuvants, functioning as innate immune trainers, bridge activation of pattern recognition receptors and metabolic reprogramming, offering a template for synthetic vaccine design [42]. The capacity of β-glucans to simultaneously activate innate immunity and modulate adaptive responses, through enhanced antigen presentation by trained macrophages and DCs, positions them as natural reprogrammers of the innate–adaptive interface [3,7,41].

Botanical and fungal polysaccharides can modulate macrophage function and APC conditioning [43,44]. Their immune effects may be useful for innate gating but are complicated by structural heterogeneity, batch variability, and context-dependent outcomes [44]. Furthermore, β-glucan has been shown to reverse the epigenetic state of LPS-induced immunological tolerance, a phenomenon of particular relevance in sepsis and chronic inflammatory conditions, in which monocytes become refractory to further stimulation [45]. By reinstating the activation of histone marks at silenced promoters, β-glucan effectively reboots the innate immune system, restoring its capacity to communicate with and activate adaptive effectors [7,45]. However, the ability of β-glucan to reverse endotoxin-induced tolerance should not be interpreted as uniformly beneficial. Breaking a tolerized myeloid state may restore antimicrobial or antitumor immune competence, but excessive or poorly localized activation could also amplify inflammatory cytokine production, sustain chronic myeloid priming, worsen tissue injury, or contribute to unintended immunopathology. This concern is particularly relevant in patients with pre-existing autoimmune or autoinflammatory disease, chronic inflammatory states, sepsis recovery, metabolic inflammation, or tissue environments where excessive IL-1β, TNF, IL-6, or inflammasome activity may be harmful. In oncology, excessive systemic activation may also increase toxicity without improving antigen-specific immunity if innate stimulation is not coordinated with antigen presentation and T-cell priming. Therefore, β-glucan-based immune reprogramming requires careful dose optimization, temporal control, compartment-specific delivery, and monitoring of inflammatory biomarkers. Preclinical evaluation should include both efficacy endpoints, such as restoration of antigen presentation and T-cell activation, and safety endpoints, including cytokine amplification, tissue inflammation, autoimmunity-related markers, and durability of the trained state after withdrawal of stimulation. This dual capacity, to both enhance naïve innate responses and rescue tolerized states, makes β-glucans uniquely versatile tools for immune reprogramming.

### 3.2. Microbial Metabolites: Short-Chain Fatty Acids and Context Stabilization

SCFAs (acetate, propionate, butyrate) regulate immunity through GPCR signaling and epigenetic mechanisms, thereby influencing T-cell differentiation and barrier immune homeostasis. They can support regulatory programs, often by enabling Treg-favoring environments, while also shaping innate responsiveness depending on tissue, dose, and timing. SCFA-like signaling often works best when localized to mucosal surfaces and when it stabilizes barrier tone rather than driving systemic activation. In immunoscape engineering, SCFAs serve as context stabilizers rather than conventional adjuvants [46,47,48].

### 3.3. Tryptophan Metabolites and Mucosal Antigen-Presenting Cell Tone

Tryptophan metabolites produced by commensals can engage sensing pathways such as aryl hydrocarbon receptor (AhR)-linked programs that regulate mucosal immunity, barrier defense, and inflammatory setpoints. This also matters for cancer, as mucosal barrier status and systemic inflammatory tone can modulate myeloid setpoints and therapy tolerability [49,50]. Microbiome-linked tryptophan metabolites and mucosal APC tone highlight how metabolic context shapes the immune landscape, connecting the microbial environment to systemic immune responses.

### 3.4. Polyphenols: Curcumin, Resveratrol, and Beyond

Plant-derived polyphenols represent another major class of natural biologics with significant immunomodulatory potential. Curcumin, the principal bioactive compound in turmeric (*Curcuma longa*), exhibits pleiotropic immunomodulatory effects, including the suppression of NF-κB signaling, inhibition of NLRP3 inflammasome activation, and modulation of T cell differentiation [29,51]. Curcumin has been shown to promote the expansion of regulatory T cells while suppressing Th1 and Th17 responses, suggesting a capacity to recalibrate the innate–adaptive balance toward tolerance in autoimmune and hyperinflammatory contexts [29]. Curcumin can modulate macrophage and DC signaling; experimental data also report effects on dendritic cell maturation and downstream T-cell proliferation [51,52]. The translational barriers to curcumin’s clinical use are bioavailability and reproducible dosing: nanocarriers can convert plausible immunomodulation into controlled exposure and combination logic (antigen plus immunomodulator) [52,53].

However, the clinical translation of polyphenol-based immunomodulation has been hampered by the notoriously poor bioavailability and rapid systemic clearance of these compounds [50,54]. Curcumin, for instance, undergoes extensive first-pass metabolism, resulting in plasma concentrations far below those required for immunomodulatory activity in vitro [50]. This pharmacokinetic limitation has spurred the development of nanoformulation strategies, including polymeric micelles, liposomes, and lipid nanoparticles, designed to enhance polyphenol stability, bioavailability, and targeted delivery to immune cells [54,55]. Several nanocarrier classes have been investigated to improve polyphenol delivery, but their translational readiness varies substantially. Liposomes offer biocompatibility, an aqueous core, a lipid bilayer, and strong clinical familiarity; however, they may suffer from leakage, limited long-term stability, oxidation of lipid components, and relatively high manufacturing costs. Polymeric nanoparticles, including PLGA-based systems, can provide sustained release and protection against degradation, but their translation depends on reproducible control of particle size and residual solvents, polymer degradation profiles, and scalable manufacturing. Polymeric micelles are useful for solubilizing hydrophobic polyphenols such as curcumin and resveratrol, although dilution after systemic administration, premature drug release, and variable stability remain concerns. Solid lipid nanoparticles and nanostructured lipid carriers offer improved physical stability and compatibility with hydrophobic compounds, but drug loading, polymorphic lipid transitions, and batch-to-batch reproducibility require careful control. Lipid nanoparticles are clinically familiar from RNA delivery applications and can improve cellular delivery, but their intrinsic immunogenicity, excipient-related effects, and repeated-dose safety must be considered. Nanoemulsions may enhance oral absorption and mucosal delivery, but they can be sensitive to surfactant composition, gastrointestinal conditions, and storage stability. More complex systems, including dendrimers, mesoporous silica nanoparticles, and extracellular vesicle-inspired carriers, may improve loading, targeting, or biological recognition, but they are generally less clinically mature and face greater challenges in toxicity testing, standardized characterization, large-scale production, and regulatory acceptance. Therefore, polyphenol nanoformulation should be selected based on the intended route, immune target, release profile, safety requirements, and clinical development pathway, rather than on bioavailability enhancement alone (Table 1). The integration of polyphenol biologics with nanomaterial delivery systems thus represents a natural point of convergence between the two pillars of this review.

### 3.5. Extracellular Vesicles and Exosomes

Extracellular vesicles (EVs), particularly exosomes (30–150 nm diameter), have emerged as endogenous natural biologics with intrinsic immunomodulatory functions [31,32]. EVs have dual attributes in this framework. They are natural biologics because their native lipid membranes, surface proteins, metabolites, and nucleic acid cargo can directly regulate immune cell communication. At the same time, they display nanocarrier-like properties because they are nanoscale vesicles capable of protecting and transporting molecular cargo between cells, crossing biological barriers, and being engineered, loaded, or hybridized for therapeutic delivery. Therefore, EVs occupy an intermediate category between biologically active immune signals and delivery platforms, which makes them particularly relevant to the convergence of natural biologics and smart nanomaterials. Exosomes derived from immune cells carry a cargo of proteins, lipids, and nucleic acids that reflect the activation state and phenotype of their parent cells, enabling them to serve as intercellular messengers that coordinate immune responses across tissue compartments [32,56]. EVs regulate immune responses by transferring proteins, lipids, and RNAs across cell membranes and play roles in both immune activation and immune suppression, depending on source and context [57,58,59]. EVs blur the line between natural biologic and nanocarrier, but face challenges in heterogeneity, scalability, and standardized characterization [57,58,59].

**Figure 3:**
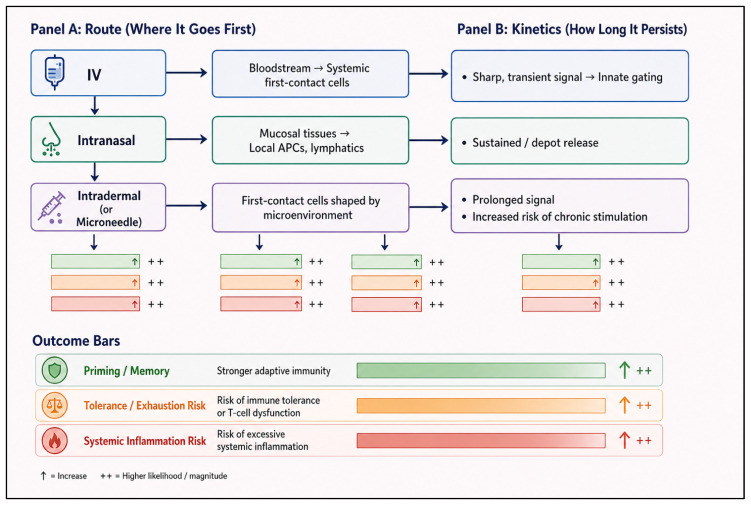
Spatiotemporal programming: route and kinetics determine immune fate. Route and release kinetics control which immune compartment encounters cues first, whether material reaches lymph nodes, and how long APCs present antigen, thereby biasing outcomes toward priming/memory, tolerance, or systemic inflammation.

Dendritic cell-derived exosomes (dexosomes) carry MHC–peptide complexes, co-stimulatory molecules, and immunomodulatory cytokines that can directly activate T cells or transfer antigenic information to bystander DCs, thereby amplifying adaptive immune responses [55]. M1 macrophage-derived exosomes have been shown to reprogram tumor-associated macrophages (TAMs) from the immunosuppressive M2 phenotype to the pro-inflammatory M1 state, effectively reshaping the tumor immune microenvironment [60,61]. These exosome-mediated reprogramming events occur through the transfer of pro-inflammatory microRNAs (miR-155, miR-125b) and the activation of NF-κB signaling in recipient cells [32,59]. Table 2 summarizes the principal natural biologic classes, their dominant immune-interface targets, and the delivery logic needed to improve translational control. Figure 3 illustrates how the delivery route and release kinetics influence first-contact immune cells, lymph node access, and downstream immune fate.

## 4. Smart Nanomaterials as Context Controllers for Immune Reprogramming

Smart nanomaterials reshape immunoscapes by controlling where cues go, how long they persist, and which cells first interpret them. This is especially valuable in cancer, where systemic dosing can cause off-target inflammation, whereas locally targeted, tumor- or node-targeted, or other spatially restricted approaches can improve the therapeutic index [2,29]. The mechanistic basis for nanomaterial-mediated immune reprogramming extends beyond simple cargo delivery; the platform itself can possess intrinsic immunological activity that influences the resulting immune phenotype.

### 4.1. Lipid Nanoparticles and mRNA Delivery

The clinical validation of lipid nanoparticle (LNP)–mRNA platforms in COVID-19 vaccines has catalyzed a revolution in immune engineering, demonstrating that synthetic nanomaterials can reprogram cellular function with remarkable precision and scalability [16,62]. In the context of the innate–adaptive interface, LNP–mRNA technology enables transient expression of immunomodulatory proteins, tumor antigens, or reprogramming factors directly within immune cells, thereby facilitating the in situ conversion of immunosuppressive cell phenotypes to immunostimulatory ones [16,63].

Recent advances have expanded the LNP–mRNA paradigm beyond prophylactic vaccination into therapeutic immune reprogramming. Intratumoral delivery of LNP-formulated mRNA encoding IL-21, IL-7, and 4-1BBL has been shown to induce systemic antitumor immunity by reprogramming the local immune milieu and promoting the expansion of tumor-specific T cells [64]. In a landmark clinical development, the randomized phase 2 KEYNOTE-942 trial demonstrated that mRNA-4157, a personalized cancer vaccine delivered via LNPs, combined with pembrolizumab, achieved a 2.5-year recurrence-free survival of 74.8% compared to 55.6% for pembrolizumab alone in resected stage III/IV melanoma [16,65]. Despite this clinical progress, personalized LNP-enabled cancer immunotherapy faces substantial translational barriers. Regulatory evaluation is more complex than for fixed-composition nanomedicines because each patient-specific vaccine may contain a distinct mRNA sequence set, requiring clear criteria for platform comparability, release testing, potency assessment, sterility, identity, purity, and safety. Manufacturing also remains demanding: tumor and normal tissue sequencing, neoantigen prediction, individualized mRNA synthesis, GMP-grade formulation into LNPs, quality control, batch release, and delivery to the treatment center must occur within a clinically actionable timeframe. Economic and logistical constraints are equally important, including high production costs, dependence on specialized bioinformatics and manufacturing infrastructure, cold-chain storage, coordination with immune checkpoint blockade, limited scalability across treatment centers, reimbursement uncertainty, and potential inequities in patient access. Repeated or combination dosing may further require pharmacovigilance for cumulative inflammatory toxicity, anti-excipient immune responses, altered biodistribution after prior exposure, and interactions with concurrent immunotherapy. Therefore, broad implementation of personalized nanomedicine platforms will depend not only on clinical efficacy but also on standardized manufacturing pipelines, validated potency assays, scalable regulatory frameworks, cost control, and equitable distribution models.

The mechanistic basis for LNP-mediated immune reprogramming extends beyond the encoded mRNA cargo. The ionizable lipid components of LNPs have been shown to possess intrinsic adjuvant activity, activating the STING pathway and promoting type I interferon production in DCs, thereby enhancing both innate immune activation and subsequent priming of adaptive responses [62,66]. Novel lipid chemistries have enabled organ-selective mRNA translation, with specific lipid tail structures directing LNP tropism to the spleen, liver, or lung, thereby opening the possibility of tissue-specific immune reprogramming [16,63]. LNPs are clinically validated platforms for RNA delivery, but innate activation can be an intrinsic feature of these platforms [18,67,68]. This is beneficial for vaccines but can be problematic for chronic dosing [18,69]. Platform immunogenicity must be aligned with clinical intent, controlled activation for vaccination versus minimized activation for repeat-dose therapeutics [18,67,69].

### 4.2. Lymph Node Targeting and Spatial Programming of Adaptive Immunity

Particle size and surface identity govern lymphatic uptake and nodal retention, determining which dendritic cell populations are engaged and how antigen is presented [26,69]. Nanoparticle vaccine strategies demonstrate that nodal routing can increase antigen persistence and improve humoral responses [15]. If the goal includes memory T cells or high-quality antibody responses, lymph node access should be treated as a non-negotiable design parameter [1,19,27,28]. Nanoparticles can target distinct dendritic cell populations based on size [27]. This reinforces the idea that size is not merely a pharmacokinetic variable; it alters the immunoscape by determining which APC subsets dominate early interpretation.

### 4.3. Biomimetic Nanoparticles

Biomimetic nanoparticles, which incorporate natural cell membrane components onto synthetic nanoparticle cores, represent a sophisticated strategy for immune modulation that leverages the biological functionality of natural membranes while retaining the engineering flexibility of synthetic platforms [70,71]. By coating nanoparticles with membranes derived from erythrocytes, leukocytes, platelets, or tumor cells, researchers can confer biologically encoded properties, including immune evasion, prolonged circulation, and cell-specific targeting, onto otherwise immunogenic synthetic carriers [71,72].

The application of biomimetic nanoparticles to the innate–adaptive interface has been particularly fruitful in nanovaccine development. Biomimetic nanovaccines that present multiple tumor antigens through tumor cell membrane coating can simultaneously address tumor heterogeneity and promote broad adaptive immune responses [72,73]. Advanced designs incorporate spatiotemporal control of immune activation, delivering antigens and adjuvants to specific tissue locations in a staged manner that mimics the natural progression of pathogen encounter, innate activation, and adaptive response generation [72,73,74].

### 4.4. Stimuli-Responsive and Smart Nanoarchitectures

Stimuli-responsive nanomaterials enable conditional release (pH/redox/ROS/enzyme triggers), allowing immunomodulation to occur preferentially within tumors or inflamed tissues [25,74,75]. Separating innate gating (early, localized activation) from sustained antigen presentation (longer duration, lymphoid access) can reduce systemic toxicity while preserving adaptive quality [25,74,75]. pH-responsive nanoparticles that disassemble in the mildly acidic tumor interstitium (pH 6.5–6.8) or within endosomal compartments (pH 5.0–5.5) have been designed to release immunostimulatory cargo precisely at sites of innate immune cell activation, thereby maximizing the local concentration of adjuvants while minimizing systemic toxicity [10,76]. ROS-responsive nanomaterials exploit the oxidative burst characteristic of activated macrophages and neutrophils, using the high ROS levels in inflamed tissues to trigger the controlled release of anti-inflammatory or reprogramming agents [9,77].

### 4.5. Macrophage Reprogramming as Immune Interface Repair

Macrophages are central regulators of tumor immunity and therapy response [29]. Nanoparticle strategies can reprogram tumor-associated macrophages (TAMs) toward phenotypes that support antigen presentation and T-cell infiltration, including approaches using TLR-agonist-loaded nanoparticles [45,78,79]. Macrophage reprogramming must be evaluated by function, antigen-presenting competence, support for T-cell activation, and effects on tissue remodeling, rather than solely by shifts in surface markers [29].

### 4.6. Corona-Aware Design

Corona formation controls the biological identity of nanoparticles and contributes to variability in immune recognition, cellular uptake, biodistribution, and clearance [9,23,24]. Corona-aware design should therefore include surface-chemistry optimization, standardized testing in human-relevant biofluids, and assessment of protein adsorption, opsonization, and complement activation. Corona profiling can help identify variability drivers, whereas complement-activation assays may flag infusion-related or repeat-dose risks. Understanding and controlling the biological corona is therefore a first-order consideration for nanomaterial design, as the immune system often responds to corona composition rather than to the pristine engineered surface. Smart nanomaterial platforms as context controllers at immune interfaces (as summarized in Table 3) systematically map the available platforms, the specific immune variables each can control, their strengths and liabilities, and their best-fit clinical applications. At present, biomolecular corona characterization is recognized as an important component of nanomedicine development, but there is no single universally accepted regulatory standard that defines a mandatory workflow for corona characterization across all nanomaterial platforms. Instead, corona evaluation is usually considered within broader nanomedicine characterization and safety frameworks that include physicochemical profiling, surface chemistry, particle-size distribution, stability, sterility, endotoxin testing, protein adsorption, complement activation, hemocompatibility, immunotoxicity, and batch-to-batch comparability. For clinical translation, this creates both a challenge and an opportunity. The challenge is that corona composition can vary with the biofluid used, anticoagulant conditions, incubation time, protein concentration, disease state, patient plasma composition, and analytical method, making cross-study comparisons difficult. The opportunity is that standardized corona profiling in human-relevant matrices could help predict opsonization, immune-cell uptake, complement activation-related infusion reactions, biodistribution, clearance, and inter-patient variability before clinical testing. A practical translational approach should therefore combine orthogonal physicochemical and proteomic methods, such as dynamic light scattering, nanoparticle tracking analysis, zeta-potential measurement, SDS-PAGE or quantitative proteomics, together with complement-activation and cytokine-release assays. Defining platform-specific corona fingerprints, acceptable batch-to-batch variability, and comparability criteria after manufacturing changes may improve reproducibility, regulatory confidence, and patient safety.

## 5. Convergence: Natural Biologics Meet Smart Nanomaterials

The most transformative developments in immune-interface reprogramming arise from the deliberate integration of natural biologics with smart nanomaterial platforms, creating hybrid systems that combine the evolved biological specificity of natural compounds with the engineering precision of synthetic nanotechnology [5,19,80]. This convergence addresses the complementary limitations of each approach: natural biologics often suffer from poor bioavailability, rapid clearance, and limited control over tissue-specific delivery, whereas synthetic nanomaterials may lack the biological recognition elements required for efficient engagement of immune cells [9,54].

Dendritic cells are not natural biologics or nanomaterials themselves; rather, they represent central immune-cell targets through which both classes of interventions can regulate the innate–adaptive interface. Dendritic cells integrate diverse innate signals, including PAMPs, DAMPs, cytokines, metabolites, and material-associated cues, and translate them into specific adaptive immune programs [4,21]. This position makes them important functional readouts for immune-interface reprogramming, particularly because natural biologics can tune dendritic-cell maturation, cytokine production, antigen-processing capacity, and tolerogenic or immunogenic programming, while smart nanomaterials can control how these signals reach dendritic cells through lymph-node routing, controlled release, surface biofunctionalization, antigen delivery, and co-delivery of adjuvant or regulatory cues [21,22,80].

Nanomaterial-based strategies to reprogram DC function in the TME have shown considerable promise. Antigen-capturing nanoparticles (AC-NPs) engineered to capture antigens directly from tumor cells and facilitate their delivery to adoptively transferred migratory cDC1s have been shown to promote in situ immunization, converting immunologically cold tumors into inflamed, T cell-infiltrated microenvironments [81]. However, the therapeutic performance of antigen-capturing nanoparticles may be constrained by several tumor- and patient-specific variables. Tumor antigen heterogeneity can limit the breadth of captured antigens, especially when antigen release is spatially uneven or dominated by subclonal tumor populations. Antigen-loss variants may emerge under immune pressure, reducing the durability of responses directed against a narrow antigenic repertoire. In addition, not all captured tumor material is equally immunogenic; effective therapy requires that captured antigens include relevant neoantigens or tumor-associated antigens that can be processed, loaded onto patient-compatible MHC molecules, and presented by functionally competent dendritic cells. Patient-specific immune variability further complicates this process. HLA genotype, baseline dendritic-cell abundance and maturation state, tumor mutational burden, prior chemotherapy or radiotherapy, lymphatic drainage, systemic inflammation, and the degree of tumor-associated myeloid suppression can all influence whether antigen capture leads to productive T-cell priming or ineffective antigen sequestration. Therefore, antigen-capturing nanoparticle strategies should be evaluated using functional endpoints that include antigen presentation, cross-presentation capacity, T-cell activation, clonal expansion, cytokine quality, tumor infiltration, and emergence of antigen-loss escape variants. Nanoparticle-mediated combinatorial targeting of multiple human DC subsets, including DC-SIGN+ and BDCA3+ populations, has led to significantly enhanced T cell activation compared with targeting either subset alone, with this enhancement depending on DC-mediated IL-15 release and intercellular DC crosstalk [82].

Natural biologics also exert their immunomodulatory effects largely by modulating DCs. β-Glucan-trained macrophages exhibit enhanced antigen-presenting capacity, and β-glucan can directly promote DC maturation and cytokine production through Dectin-1 signaling [7,37,42]. Polyphenols such as curcumin modulate DC function by suppressing pro-inflammatory cytokine production and enhancing the expression of tolerogenic markers, thereby providing a pathway for the induction of immune tolerance in autoimmune disease [29,51]. The convergence of these natural biological effects with engineered nanomaterial delivery creates a powerful toolkit for the precise manipulation of DC-mediated innate–adaptive crosstalk [19,83].

Related pharmacological and nanomedicine studies further support this logic of convergence. Teriflunomide-mediated suppression of T helper cells and dendritic cells alleviates experimental autoimmune uveitis, whereas immunoconjugate-based nanocomplexes that integrate chemotherapy with immune checkpoint therapy synergistically improve therapeutic efficacy in small-cell lung cancer [84,85].

**Figure 4:**
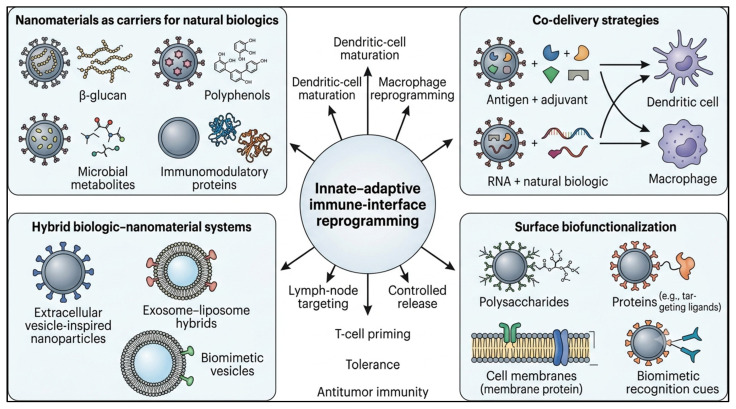
Combined application modes of natural biologics and smart nanomaterials for immune-interface reprogramming. Natural biologics and smart nanomaterials can converge through four main translational modes: nanomaterials as carriers for natural biologics; co-delivery of natural biologics with antigens, adjuvants, RNA, or small-molecule payloads; incorporation of biologically derived components as surface biofunctionalization cues; and hybrid biologic–nanomaterial systems, including EV-inspired nanoparticles, exosome–liposome hybrids, and biomimetic vesicles. These strategies connect biological immune conditioning with engineered control over biodistribution, cellular targeting, release kinetics, and safety.

The combined application of natural biologics and smart nanomaterials can be organized into four practical modes. First, nanomaterials can serve as delivery systems for natural biologics, such as β-glucans, polyphenols, microbial metabolites, nucleic-acid-containing vesicles, or immunomodulatory proteins, thereby improving solubility, stability, biodistribution, lymphatic access, cellular uptake, and release kinetics. Second, natural biologics can be used as immune-conditioning agents co-delivered with antigens, adjuvants, RNA, or small-molecule payloads to tune dendritic cell maturation, macrophage polarization, trained immunity states, or tolerogenic programs. Third, biologically derived components can be incorporated into nanomaterial surfaces to provide recognition cues, immune camouflage, adhesion signals, or APC-targeting properties, as seen in cell-membrane-coated nanoparticles, biomimetic vesicles, and protein- or polysaccharide-functionalized carriers. Fourth, hybrid biologic–nanomaterial systems can combine biological signaling with engineered control, including extracellular vesicle-inspired nanoparticles, exosome–liposome hybrids, and biomimetic carriers that use natural membrane components while retaining synthetic control over size, loading, release, and scalability. These modes show that the relationship between natural biologics and nanomaterials is not limited to simple cargo encapsulation; rather, it includes immune conditioning, surface biofunctionalization, biomimicry, and integrated control of the innate–adaptive interface. Figure 4 summarizes these four combined application modes and illustrates how natural biologics and smart nanomaterials can be integrated to regulate innate–adaptive immune-interface outcomes.

These combined application modes are summarized schematically in Figure 4, which illustrates how natural biologics and smart nanomaterials can converge through cargo delivery, co-delivery, surface biofunctionalization, and hybrid biologic–nanomaterial systems to regulate immune-interface outcomes.

A critical distinction should be made between biomimetic nanoparticles and ligand-targeted nanoparticles. Biomimetic systems, such as cell-membrane-coated particles, tumor-membrane nanovaccines, platelet-mimetic carriers, leukocyte-mimetic particles, and extracellular vesicle-inspired systems, can provide complex natural recognition cues, immune evasion signals, multivalent surface proteins, and biologically matched adhesion molecules. These features may improve circulation, tissue interactions, or immune cell communication. However, their targeting specificity is often less well defined than that of ligand-targeted platforms because the membrane coating contains heterogeneous, variable biological components. Biomimetic systems also face major translational challenges, including standardization of membrane sources, donor or cell-line variability, safety of pathogens and oncogenic signals, batch-to-batch comparability, large-scale membrane isolation, preservation of membrane orientation, and regulatory characterization of a complex, biologically derived surface. In contrast, ligand-targeted nanoparticles use defined targeting moieties, such as antibodies, antibody fragments, peptides, aptamers, carbohydrates, or small molecules, to engage selected receptors. These systems may offer clearer definitions of the mechanism of action, more controllable surface chemistry, and easier analytical characterization, but their efficacy can be limited by receptor heterogeneity, target downregulation, ligand-density optimization, steric hindrance, off-target binding, ligand immunogenicity, and accelerated clearance. Therefore, biomimetic nanoparticles should not be viewed as universally superior to ligand-targeted nanocarriers. Instead, they are best suited for applications requiring multivalent biological recognition or immune camouflage, whereas ligand-targeted systems may be preferable when a validated receptor target, scalable chemistry, and regulatory simplicity are prioritized (Table 4).

## 6. Integrative Design Rules for Reprogramming Immunoscapes

The immunoscape framework implies that context frequently dominates cargo. The same biologic can become immunogenic, tolerogenic, or toxic depending on route, kinetics, corona identity, and the first cell of contact [2,4,9,23,24,27,28]. These observations translate into actionable design principles for nanomaterial-based immune reprogramming.

Rule 1: Context Beats Cargo. Route and kinetics often decide the outcome [1,19,25,74]. The physicochemical properties of nanomaterials, size, surface charge, lipid composition, and release kinetics can transform a single biologic into either an immunogenic stimulus or a tolerogenic signal, depending on which tissue compartment encounters it first and with what temporal dynamics.

Rule 2: Program the First Interpreters. Early myeloid and APC gating determine adaptive fate; tumor macrophages are especially decisive in cancer [29]. Before designing the adaptive phase of an immune response, carefully consider which innate compartment will encounter the therapeutic payload initially and how its phenotype influences downstream adaptive programming.

Rule 3: Use Sequential Logic. Separate early gating (localized activation/reprogramming) from longer presentation/priming phases [25,74]. This design principle enables efficient engagement and reprogramming of innate cells without imposing the sustained inflammatory burden of continuous systemic innate activation.

Rule 4: Benchmark Function, Not Markers. Measure antigen presentation competence, T-cell quality, and memory durability [2,29]. Surface marker changes (CD80, CD86, M1/M2 markers) do not reliably predict functional outcomes; functional assays that measure actual antigen presentation, cytotoxic T cell priming, and memory formation are essential. A standardized preclinical assay panel for immune-reprogramming nanomaterials should therefore combine phenotypic, functional, and safety-oriented readouts. First, innate activation should be assessed using cytokine and chemokine profiling, including IL-1β, IL-6, TNF, IL-12, type I interferons, CXCL10, and relevant inflammasome-associated mediators. Second, antigen uptake, processing, and presentation should be measured using antigen internalization assays, MHC-I and MHC-II peptide-presentation assays, cross-presentation assays, and co-culture systems with antigen-specific CD8+ and CD4+ T cells. Third, dendritic cell and macrophage function should be evaluated by maturation status, phagocytosis, antigen-presenting competence, metabolic rewiring, and the ability to support T-cell activation, rather than by CD80/CD86 or M1/M2 marker shifts alone. Fourth, adaptive immune quality should be tested through T-cell proliferation, cytotoxicity, cytokine polyfunctionality, regulatory T-cell induction, exhaustion markers, memory differentiation, and, where relevant, B-cell activation and antibody class switching. Fifth, safety and translational compatibility should be assessed by evaluating complement activation, hemolysis, platelet activation, coagulation effects, cytokine release risk, inflammasome activation, cell viability, tissue inflammation, and repeat-dose responses. Finally, the durability and reversibility of immune reprogramming should be examined using longitudinal restimulation assays, withdrawal studies, and comparisons across human donor cells to capture inter-individual variability.

**Figure 5:**
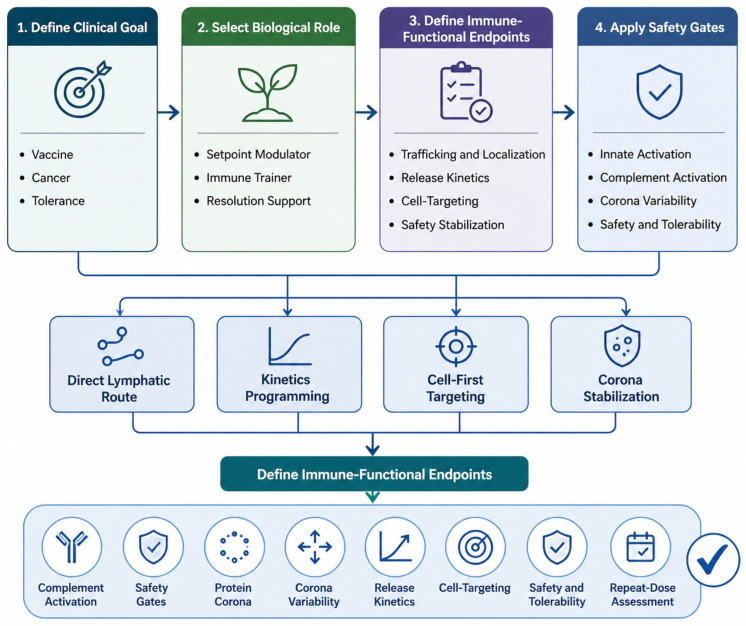
Co-delivery logic and sequential signaling: a design algorithm for immune interface reprogramming. Translation-oriented decision algorithm linking clinical intent to biologic function, nanomaterial context control, measurable immune outcomes, and early safety gates.

Rule 5: Safety is a Design Parameter. Corona and platform immunogenicity shape complement, clearance, and repeat-dose risk [4,9,23,24]. From the earliest stages of nanomaterial design, anticipate corona formation and platform-intrinsic immunogenicity, and incorporate design features that minimize repeat-dose liabilities. Figure 5 summarizes the co-delivery and sequential-signaling logic needed to combine natural biologics with engineered nanomaterial platforms. Table 5 links nanomaterial design variables to expected immune phenotypes and translational risks.

## 7. Evaluation Framework for Translation

A translationally useful design framework must articulate not only what exists and how to design, but what to measure and how to manage safety and variability. Early-phase evaluation should be comprehensive, addressing both the intended immunological effects and potential liabilities.

### 7.1. Innate Gating and Immunotoxicity Screens

**Figure 6:**
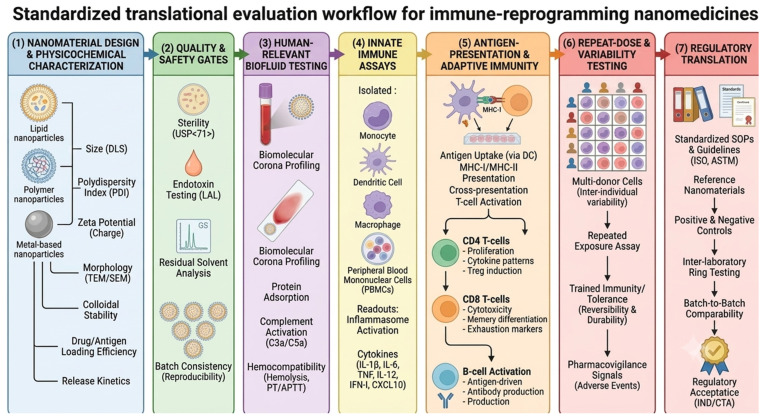
Standardized translational evaluation workflow for immune-reprogramming nanomedicines. A practical preclinical evaluation pipeline should combine physicochemical characterization, endotoxin and sterility control, biomolecular corona profiling, complement activation testing, cytokine and chemokine profiling, antigen uptake and presentation assays, dendritic-cell and macrophage functional assessment, T-cell and B-cell response evaluation, hemocompatibility testing, repeat-dose assessment, and multi-donor human immune-cell validation. Harmonized protocols, reference nanomaterials, standardized human biofluids, predefined dose metrics, positive and negative controls, inter-laboratory comparison, and batch-comparability criteria are needed to improve reproducibility, comparability, and regulatory confidence.

Early-phase evaluation should define the innate gate that the system establishes, as this gate determines APC fate and downstream adaptive quality [3,5,21,22]. Key assays include dose- and time-resolved cytokine signatures and viability to distinguish controlled gating from uncontrolled inflammation. Complement and corona are central translational variables. Corona profiling can identify variability drivers; complement activation assays can flag infusion or repeat-dose risks [9,23,24]. The proposed standardized translational evaluation workflow is summarized in Figure 6. This workflow integrates physicochemical characterization, sterility and endotoxin control, biomolecular corona profiling, complement activation testing, cytokine and chemokine profiling, antigen uptake and presentation assays, dendritic cell and macrophage functional assessment, adaptive immune response evaluation, hemocompatibility testing, repeat-dose assessment, and multi-donor human immune cell validation. By combining these assays with harmonized protocols, reference nanomaterials, standardized human biofluids, predefined dose metrics, validated controls, inter-laboratory comparison, and batch-comparability criteria, this framework is intended to improve reproducibility, cross-study comparability, and regulatory confidence during preclinical development.

Standardization of these assays will require harmonized protocols rather than isolated endpoint selection. For cytokine profiling, laboratories should use predefined immune-cell sources, such as human PBMCs, monocyte-derived dendritic cells, macrophages, or whole-blood models, with standardized donor numbers, stimulation times, dose metrics, viability thresholds, endotoxin limits, and positive and negative controls. Cytokine panels should include both inflammatory and regulatory mediators and should be reported with information on assay platform, lower limit of detection, normalization method, and donor-to-donor variability. For complement activation testing, standardized human serum or plasma conditions, anticoagulant choice, incubation time, temperature, nanoparticle concentration, and readouts such as C3a, C5a, sC5b-9, CH50/AH50, and hemolysis should be prespecified. For corona analysis, biofluid source, protein concentration, incubation conditions, washing procedure, separation method, and analytical platform should be reported in detail, ideally using orthogonal methods such as DLS or nanoparticle tracking analysis, zeta-potential measurement, SDS-PAGE, LC–MS/MS proteomics, and complement or opsonization assays. Across all three domains, reproducibility would be strengthened by reference nanomaterials, shared control materials, inter-laboratory ring studies, standardized reporting templates, and predefined batch-comparability criteria. Regulatory acceptance will further require assay validation for sensitivity, specificity, precision, robustness, reproducibility, and relevance to clinically observed immune toxicity or efficacy.

### 7.2. Antigen-Presenting Cell Function and Antigen Presentation Quality

Because APCs are the bridge to adaptive immunity, functional assays should prioritize antigen uptake/processing, cross-presentation competence, and migratory capacity to lymph nodes [1,19,27]. In cancer, APC function should be evaluated under tumor-like constraints (immunosuppressive cytokines and, when feasible, hypoxia-mimicking conditions), because the tumor immunoscape can invert what appears effective in simplified assays [29].

### 7.3. Adaptive Quality Endpoints

Adaptive endpoints should emphasize quality, not only magnitude: polyfunctionality, memory durability, and avoidance of exhaustion/tolerance phenotypes [2,29]. In vaccine paradigms, humoral quality metrics (neutralization and affinity maturation proxies) are central; in cancer, durable cytotoxicity and competence in tumor infiltration are central [2,29].

### 7.4. Biodistribution and Lymphoid Access

Spatial access should be demonstrated: lymph node accumulation when LN priming is required, or tumor localization when TAM reprogramming is intended [1,19,27,28].

### 7.5. Platform-Specific Liabilities

**Figure 7:**
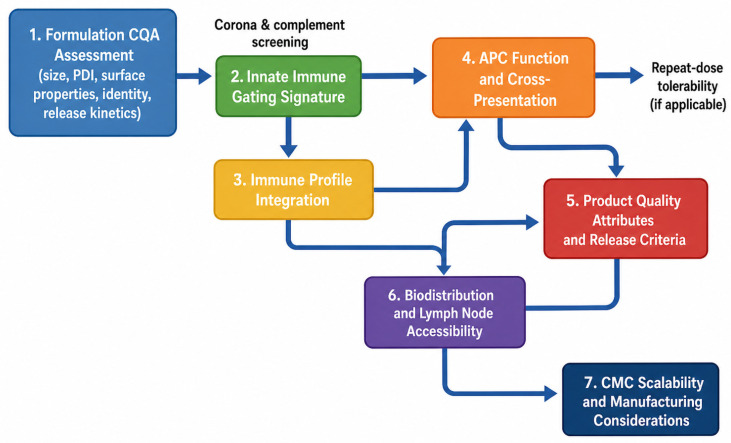
Translation pipeline. Practical workflow connecting nanomaterial critical quality attributes to mechanistic immune benchmarks, integrating corona-aware immunotoxicity screening and repeat-dose risk management.

For LNPs and other immune-active platforms, reactogenicity and innate activation must be evaluated under the intended dosing schedules, as chronic dosing changes the risk-benefit calculus [17,18,67,68]. The minimal evaluation panel for immune-reprogramming nanomedicines (as summarized in Table 6) consolidates the key assays, primary endpoints, and operational pass criteria for translating nanomaterial-based immune-reprogramming strategies. Figure 7 presents a stepwise translational evaluation framework for smart nanomaterial-based immune reprogramming. The workflow begins with the assessment of formulation critical quality attributes, including particle size, polydispersity index, surface properties, identity, and release kinetics. These parameters are essential because they influence biodistribution, biological identity, immune-cell uptake, and reproducibility. The next stage involves corona and complement screening, followed by evaluation of the innate immune-gating signature, which helps determine whether the formulation induces acceptable early immune activation or poses a risk of excessive inflammatory responses. The framework then integrates immune profiling with antigen-presenting cell function and cross-presentation assays, linking early innate sensing to adaptive immune competence. Importantly, the figure also emphasizes repeat-dose tolerability, product quality attributes, release criteria, biodistribution, lymph node accessibility, and chemistry, manufacturing, and control scalability. Together, these checkpoints provide a practical roadmap for moving candidate nanomaterial–biologic platforms from mechanistic design toward translational development, while maintaining safety, reproducibility, and clinical feasibility.

## 8. Clinical Translation and Emerging Applications

The translational roadmap for immunoscape engineering benefits from explicitly matching the biologic and nanomaterial roles to a clinical goal. A key milestone in translating nanomaterial-based immune reprogramming is the emergence of clinically validated platforms and active clinical trials demonstrating the approach’s viability across multiple disease contexts.

### 8.1. Cancer Immunotherapy

The most advanced clinical applications of innate–adaptive interface reprogramming lie in oncology, where the failure of conventional immunotherapies in a substantial proportion of patients has driven the search for strategies that address both innate and adaptive immune deficits within the TME [16,34,35]. The TME presents a uniquely challenging immune landscape, characterized by the coexistence of immunosuppressive macrophages, dysfunctional DCs, exhausted T cells, and regulatory cell populations that collectively enforce immune evasion [34,35].

Cancer immunoscapes often include TAM-dominated suppression, impaired antigen presentation, and dysfunctional T-cell states [29]. Nanomaterials can intervene at two critical levels: innate interface repair through TAM reprogramming to restore antigen presentation and reduce suppression [29,46], and adaptive programming by ensuring that antigens and cues reach lymphoid tissues and are presented with appropriate kinetics to support durable T-cell programs [1,19,27]. A key strength of nanomedicine is the ability to combine these approaches: localized TAM programming, stimuli-responsive release, and lymph node routing for sustained adaptive instruction [25,74].

### 8.2. Infectious Disease and Vaccine Development

Beyond oncology, the reprogramming of the innate–adaptive interface has significant implications for infectious disease prevention and treatment. The trained immunity paradigm offers a conceptual framework for the development of next-generation vaccines that engage both innate memory and adaptive specificity [2,3,28]. BCG-induced trained immunity, which enhances heterologous protection against unrelated pathogens, has been proposed as a basis for universal immunity boosting strategies, particularly in neonatal and immunocompromised populations [28].

### 8.3. Autoimmune Disease and Transplantation

The bidirectional nature of immune reprogramming, capable of both enhancing and suppressing immune responses depending on the biologic or nanomaterial employed, creates opportunities for therapeutic applications in autoimmune disease and transplant immunology [29,83]. Tolerogenic nanoparticles loaded with autoantigens and immunosuppressive biologics (such as curcumin, rapamycin, or IL-10) have been designed to reprogram DCs toward a tolerogenic phenotype, promoting the expansion of antigen-specific regulatory T cells while suppressing pathogenic effector responses [29,52,86].

### 8.4. Tissue Regeneration and Wound Healing

The innate–adaptive immune interface plays a critical role in tissue repair and regeneration, with the sequential activation and resolution of inflammatory responses governing the transition from tissue damage to functional restoration [76,86]. Macrophage phenotype switching, from pro-inflammatory M1 to pro-regenerative M2, is a key determinant of regenerative outcomes, and dysregulation of this transition underlies pathological fibrosis and chronic wound-healing failure [34,86].

### 8.5. Approved Nanomedicines and Active Trials

The immunoscape concept is not theoretical; several approved nanomedicines already demonstrate that controlling where and how bioactive signals are delivered can yield real-world benefit. A key milestone was patisiran (ONPATTRO^®^), the first FDA-approved lipid nanoparticle (LNP) siRNA therapeutic (approved in 2018) [87,88], which established the regulatory and clinical feasibility of LNP-enabled nucleic acid delivery. This approval validated the principle that nanomaterial platforms can safely deliver nucleic acids to achieve therapeutic effects in humans. An mRNA-based COVID-19 vaccine indicated for active immunization against SARS-CoV-2, with the 2025–2026 formula approved for individuals aged 65 years and older and for those aged 5–64 years with at least one underlying condition that increases the risk of severe COVID-19 outcomes. The prescribing information also highlights key safety considerations for mRNA–LNP platforms, including postmarketing evidence of increased risks of myocarditis and pericarditis, particularly within the first week after vaccination and most prominently in males aged 12–24 years [89].

Clinically used natural biologic immunotherapy: BCG provides a long-standing model of localized innate–adaptive reprogramming. Intravesical BCG (e.g., TICE^®^ BCG) is an FDA-labeled immunotherapy used for carcinoma in situ (CIS) of the urinary bladder and for prophylaxis against recurrence of certain high-risk non-muscle-invasive bladder tumors [90]. Guidelines from urologic societies include intravesical BCG as a core element of risk-adapted NMIBC management, reinforcing the notion that localized innate activation can therapeutically reshape the immunoscape in a clinically durable manner [91]. Clinically used immunoscape-modulating interventions and representative active trials (as summarized in Table 7) provide a comprehensive overview of approved nanomedicines and active clinical trials that demonstrate the clinical translation of immune interface reprogramming strategies.

## 9. Conclusions

The innate–adaptive immune interface, once viewed as a static relay between two discrete systems, is now recognized as a dynamic, bidirectional, and reprogrammable frontier that determines the trajectory of immune responses in health and disease. The convergence of natural biologics and smart nanomaterials has created an unprecedented toolkit for therapeutic modulation of this interface, enabling researchers and clinicians to reshape immune programs through coordinated epigenetic marks, metabolic shifts, antigen-presenting cell fate decisions, and molecular signaling networks.

Natural biologics, from β-glucans that induce trained immunity through epigenetic rewiring of myeloid progenitors, to polyphenols that recalibrate T-cell polarization, exosomes that deliver intercellular reprogramming cargo, and short-chain fatty acids and tryptophan metabolites that stabilize immunological setpoints, provide the biological vocabulary for immune modulation [2,7,29,31,47,48]. Smart nanomaterials, including LNPs that enable in vivo expression of reprogramming factors, biomimetic nanoparticles that mimic natural immune signaling, stimuli-responsive platforms that achieve tissue-specific activation, and corona-aware designs that improve reproducibility, provide the structural and delivery logic that organizes these biological signals into coherent therapeutic programs [9,23,63,71,77].

Dendritic cells, as central translators of the innate–adaptive interface, serve as a primary cellular substrate for these reprogramming strategies. By integrating natural biologic signals with nanomaterial-delivered instructions, dendritic cells can direct adaptive immune responses toward activation, tolerance, memory formation, or antitumor immunity, depending on the clinical objective [21,22,81]. The clinical validation of LNP–mRNA platforms, together with the growing recognition of trained immunity as a therapeutically exploitable process, signals the beginning of a new era in immune-interface engineering [16,66].

Looking forward, several priorities should guide the next phase of research. First, future studies should move beyond descriptive immunomodulation and establish mechanism-based design rules linking nanomaterial properties, biologic cargo, route of administration, release kinetics, and immune outcomes. Particular attention should be given to the “first-contact” immune cells that initially interpret therapeutic materials, as these early cellular interactions can determine whether downstream adaptive responses become protective, tolerogenic, exhausted, or inflammatory. Second, greater emphasis is needed on human-relevant models, including primary immune cell systems, organoids, lymph node-on-chip platforms, tumor–immune co-cultures, and ex vivo patient-derived assays, to bridge the gap between preclinical promise and clinical predictability. Third, future work should prioritize the standardized evaluation of biomolecular corona formation, complement activation, innate immune gating, antigen-presenting cell function, cross-presentation, biodistribution, lymph node accessibility, and repeat-dose tolerability. These parameters should not be treated as secondary safety checks but as core elements of rational immunotherapeutic design.

In addition, future directions should include the development of personalized immunoscape profiling, where patient-specific inflammatory status, microbiome-derived metabolites, tumor immune phenotype, plasma protein corona patterns, and baseline trained-immunity signatures are used to guide therapeutic selection and dosing. Artificial intelligence and computational modeling may further accelerate this process by predicting how material composition, cargo combinations, and delivery routes influence immune trajectories. Such approaches could support the design of adaptive nanomedicine platforms that are not only targeted but also context-aware and patient-responsive.

The immunoscape framework, therefore, provides more than a unifying conceptual model; it offers practical design rules: context beats cargo, program the first interpreters, use sequential logic, benchmark immune function, and treat safety as a design parameter. It also supports an evaluation pipeline that integrates efficacy with corona variability, complement risk, manufacturing reproducibility, and repeat-dose safety. This translational emphasis can guide clinically realistic immune programming for cancer immunotherapy, vaccines, inflammatory disorders, regenerative medicine, and the induction of immune tolerance.

As the field advances, the major challenges will include manufacturing scalability, batch-to-batch reproducibility of natural biologics, long-term immunotoxicity, regulatory classification of hybrid biologic–nanomaterial platforms, and the need for clinically meaningful biomarkers of immune reprogramming. Nevertheless, the potential to reprogram immunity at its most fundamental interface offers a transformative vision for the future of medicine. The immunoscapes ahead remain partly uncharted, but interdisciplinary integration of immunology, materials science, bioengineering, systems biology, and computational design is beginning to reveal their contours and may ultimately enable safer, more precise, and durable immune therapies.

## Figures and Tables

**Table 1:** Comparative translational features of nanocarrier systems for polyphenol delivery.

Nanocarrier System	Main Advantages	Main Limitations	Relative Clinical Readiness
Liposomes	Biocompatible; clinically familiar; suitable for hydrophilic and hydrophobic cargo; can improve stability and tissue exposure	Drug leakage; lipid oxidation; limited long-term stability; relatively high manufacturing cost	High
Polymeric nanoparticles, including PLGA-based systems	Sustained release; protection from degradation; tunable size, charge, and degradation profile	Residual-solvent concerns; polymer degradation variability; scale-up and batch reproducibility challenges	Moderate
Polymeric micelles	Efficient solubilization of hydrophobic polyphenols such as curcumin and resveratrol; small size; useful for systemic delivery	Dilution instability after administration; premature drug release; variable circulation stability	Moderate
Solid lipid nanoparticles and nanostructured lipid carriers	Useful for hydrophobic cargo; improved physical stability; compatible with oral, topical, and parenteral delivery	Limited drug loading in some formulations; lipid polymorphism; risk of drug expulsion during storage; reproducibility issues	Moderate
Lipid nanoparticles	Strong cellular delivery performance; clinically familiar platform class; tunable lipid composition and tissue tropism	Intrinsic immunogenicity; excipient-related effects; complement activation; repeated-dose safety concerns	Moderate to high, depending on indication
Nanoemulsions	Improved oral or mucosal absorption; scalable production potential; suitable for poorly soluble compounds	Surfactant sensitivity; gastrointestinal instability; storage instability; possible irritation depending on excipients	Moderate
Dendrimers	High loading capacity; multivalent surface functionalization; potential for ligand-based targeting	Toxicity concerns; complex synthesis; difficult regulatory characterization; limited clinical maturity	Low to moderate
Mesoporous silica nanoparticles	High surface area; controllable pore size; high loading capacity; surface can be functionalized	Biodegradation and long-term safety concerns; possible inflammatory responses; regulatory uncertainty	Low to moderate
Extracellular vesicle-inspired carriers	Biological recognition; immune-cell communication potential; possible improved biocompatibility	Heterogeneity; difficult isolation and purification; scalability limitations; lack of standardized characterization	Low to moderate

**Table 2:** Natural biologics that reprogram innate–adaptive immune interfaces.

Class	Representative Examples	Dominant Interface Node(s)	Key Mechanisms	Typical Immune Outcome	Translational Limitations	Best-Fit Delivery Logic	Key Refs
Microbial metabolites	SCFAs (acetate/propionate/butyrate)	Mucosal tone; T-cell polarization	GPCR signaling; epigenetic modulation	Context stabilization; Treg-support in many settings	Rapid metabolism; localization needed	Oral/mucosal, controlled release	[47,48,49]
Tryptophan metabolites	Indole derivatives (microbiome-linked)	Barrier immunity; mucosal APC tone	AhR/PXR-axis linked tuning	Barrier defense; inflammatory setpoint control	Inter-individual microbiome variability	Mucosal targeting; microbiome-aware strategies	[50,51]
Trained immunity inducers	β-glucans	Innate/progenitor memory	Epigenetic + metabolic rewiring	Enhanced innate readiness	Risk of chronic inflammatory priming	Controlled kinetics; compartment targeting	[3,5,21,22]
Live attenuated cue lesson	BCG (trained immunity evidence)	Systemic innate setpoint	Durable innate functional shift	Broader innate “reset”	Context/population variability	Inform design (not “delivered” here)	[39,40]
Plant/fungal polysaccharides	Diverse botanical/fungal polysaccharides	PRR tuning; APC programming	Macrophage/APC modulation	Immunogenic or regulatory (context-dependent)	Batch heterogeneity; standardization	Encapsulation + QC + context control	[44,45]
Phytochemicals	Curcumin	Macrophage/DC programming	NF-κB/cytokine circuit tuning	Anti-inflammatory or immune tuning	Poor bioavailability; dosing control	Nanoencapsulation; co-delivery	[53,54]
Extracellular vesicles	Exosomes/EVs (various sources)	Intercellular immune messaging	RNA/protein cargo effects	Immunomodulation	Heterogeneity; scalability; standards	EV-inspired hybrids; standardized isolation	[58,59,60]

**Table 3:** Smart nanomaterial platforms according to the context-control lever they provide, their typical payloads, and their major immune liabilities.

Class	Representative Examples	Dominant Interface Node(s)	Key Mechanisms	Typical Immune Outcome	Translational Limitations	Best-Fit Delivery Logic	Key Refs
Lipid nanoparticles	Ionizable LNPs; mRNA/siRNA LNPs	Innate sensing; APC programming; tissue tropism	Endosomal delivery; transient RNA expression; platform-dependent innate activation	Controlled antigen or cytokine expression; vaccine adjuvanticity; immune reprogramming	Reactogenicity; repeat-dose inflammatory risk; PEG-lipid concerns	Tune lipid composition, dose, and route according to vaccination versus chronic therapy intent	[16,17,18,62,63,64,65,66,67,68,69]
Lymph-node-targeted nanoparticles	Albumin-binding particles; size-optimized nanovaccines	Lymphatic transport; nodal APC engagement	Particle-size- and surface-dependent lymphatic uptake and retention	Improved antigen persistence, T-cell priming, and humoral quality	Variable lymphatic access; interstitial barriers; size/PDI sensitivity	Optimize size, PDI, charge, and injection route for nodal exposure	[1,15,19,26,27,28,69]
Biomimetic nanoparticles	Cell-membrane-coated particles; tumor-membrane nanovaccines	Immune recognition; antigen breadth; circulation	Natural membrane camouflage; multivalent antigen presentation; cell-specific adhesion cues	Broader antitumor immunity and improved immune-cell interaction	Source heterogeneity; manufacturing complexity; characterization burden	Use standardized membrane sourcing, antigen profiling, and batch-release criteria	[70,71,72,73,74]
Stimuli-responsive nanoarchitectures	pH-, redox-, ROS-, and enzyme-responsive carriers	Tumor/inflamed-tissue gating; release kinetics	Conditional disassembly or cargo release in pathological microenvironments	Localized immune activation with reduced systemic toxicity	Trigger heterogeneity; incomplete release; off-target activation	Match trigger chemistry to disease microenvironment and validate release in human-relevant matrices	[9,10,25,74,75,76,77]
Macrophage/TAM-reprogramming nanoparticles	TLR-agonist-loaded particles; metabolic or cytokine-modulating carriers	Myeloid setpoint; antigen presentation; T-cell infiltration	Local innate activation, metabolic rewiring, or M2-to-M1-like repolarization	Repair of suppressive tumor interfaces and improved adaptive competence	Marker-only interpretation; inflammatory toxicity; tumor heterogeneity	Prioritize functional readouts: antigen presentation, T-cell activation, and tissue remodeling	[29,45,78,79]
Corona-aware surface-engineered nanoparticles	PEGylated, zwitterionic, protein-preconditioned, or biomolecule-coated systems	Biological identity; complement activation; clearance	Control of protein adsorption, opsonization, and complement engagement	Improved reproducibility, safety, and predictable immune-cell uptake	Patient-to-patient plasma variability; repeat-dose risks	Include standardized corona profiling and complement assays early in development	[9,23,24]

**Table 4:** Critical comparison between biomimetic nanoparticles and ligand-targeted nanoparticles for immune-interface reprogramming.

Feature	Biomimetic Nanoparticles	Ligand-Targeted Nanoparticles
Targeting principle	Natural membrane-derived recognition cues and multivalent biological signals	Defined receptor–ligand interaction
Targeting specificity	Broad biological tropism; may be context-dependent	More precise if target receptor is validated
Main advantages	Immune camouflage, prolonged circulation, multivalent recognition, biological adhesion cues	Defined mechanism, tunable ligand density, easier analytical control
Main limitations	Heterogeneous membrane composition, donor/cell-source variability, uncertain orientation of membrane proteins	Receptor heterogeneity, target loss, off-target binding, ligand immunogenicity
Scalability	More difficult because of membrane sourcing, isolation, and preservation	Generally more scalable through chemical conjugation or controlled surface modification
Manufacturing complexity	High; requires membrane extraction, coating, characterization, and sterility control	Moderate; requires reproducible conjugation and ligand-density control
Regulatory considerations	Complex biologically derived surface; greater need for identity, purity, comparability, and safety testing	More straightforward if ligand, linker, and particle chemistry are well defined
Best-fit use	Immune evasion, tumor-membrane vaccines, cell-mimetic targeting, EV-inspired delivery	Receptor-specific delivery, precision targeting, scalable nanomedicine design

**Table 5:** Design variables linking material properties and immune phenotypes.

Design Variable	Typical Biological Consequence	Interface Node Affected	Preferred Direction (Examples)	Practical Strategy	Go/No-Go Readout	Key Refs
Size	LN routing vs. peripheral retention; APC subset selection	Spatial priming; APC sequence	Vaccines/cancer vaccines: favor LN access; Tumor-local: favor local retention	Size optimization; stable PDI	LN accumulation or local tumor uptake with function	[1,19,27,28]
Surface chemistry	Corona + opsonization variability	Uptake; complement	Repeat-dose: reduce variability; Vaccines: controlled activation	PEG/zwitterions; corona testing	Complement activation + uptake predictability	[9,23,24]
Release kinetics	Pulse vs. sustained cues	Priming vs. tolerance/exhaustion	Sequential logic for safe priming	Stimuli-responsive/depot	Adaptive quality without chronic toxicity	[25,74,75]
Platform immunogenicity	Intrinsic innate activation	Innate gating	Vaccines: moderate; Repeat-dose: minimize	LNP composition tuning	Reactogenicity + repeat-dose tolerability	[17,18,67,68]
Cell-first targeting	Which cell interprets cue first	APC fate; TAM state	Cancer: TAM/DC programming	Ligands; local delivery	Antigen presentation function ↑	[45,79]
Route	Tissue niche + lymphatic routing	Geography of instruction	Cancer: intratumoral/peritumoral when feasible; systemic when needed	Route optimization	Desired tissue/LN exposure achieved	[1,19,25]

**Table 6:** Evaluation panel for immune-reprogramming nanomedicines across innate, adaptive, biodistribution, and safety endpoints.

Domain	Assay Examples	Primary Endpoints	Operational “Pass” Criteria	Notes	Key Refs
Innate gating	Cytokine time-course; viability across dose	Controlled innate tone	No uncontrolled cytokine surge at effective dose	Timing matters; compare pulse vs. sustained	[3,5,25]
Corona & complement	Corona profiling; complement activation	Predictable immune identity	Stable corona signature; acceptable complement activation	Patient variability must be considered	[9,23,24]
APC programming	Uptake/processing; cross-presentation; migration	Antigen presentation function	Improved functional presentation vs. control	Function > surface markers	[1,19,27]
Adaptive quality	Polyfunctionality; memory durability	Durable quality response	Memory durable without exhaustion/tolerance	Match endpoint to indication	[2,29]
Biodistribution	LN accumulation; tumor retention	Spatial access achieved	LN access or tumor localization confirmed	Size/PDI and route sensitive	[1,19,28]
Repeat-dose risk	Repeat dosing innate panel	Tolerability signatures	Acceptable innate activation on repeat	Critical for chronic RNA therapy	[17,18,67,68]

**Table 7:** Clinically used immunoscape-modulating interventions and representative trials illustrating translational maturity.

Category	Intervention (Platform)	Clinical Status	Indication/Setting	Key Trial or Regulatory Anchor	Key Refs
Approved nanomedicine (RNA–LNP)	Patisiran (ONPATTRO^®^; siRNA–LNP)	Approved	hATTR amyloidosis polyneuropathy	First FDA-approved LNP-siRNA therapeutic (2018)	[85,86]
Approved nanomedicine (mRNA–LNP vaccine)	COMIRNATY^®^ (BNT162b2; mRNA–LNP)	Approved	COVID-19 prevention	FDA labeling/safety updates	[87]
Cancer immunotherapy (personalized mRNA)	mRNA-4157 (V940) + pembrolizumab	Phase 2 data; Phase 3 initiated	Resected high-risk melanoma; expansion to other tumors	KEYNOTE-942 publication; NCT03897881; phase 3 initiation announcements	[66,92,93]
Cancer immunotherapy (follow-up)	mRNA-4157 (V940) + pembrolizumab	Follow-up presentations	Melanoma (longer follow-up)	ASCO long-term update (conference report)	[66]
Cancer immunotherapy (neoantigen immunotherapy)	RO7198457 (iNeST-related) ± ICI	Active clinical testing	ctDNA-informed CRC and other solid tumors	ClinicalTrials.gov record (example)	[94]
Clinically used natural biologic immunotherapy	Intravesical TICE^®^ BCG	Approved/guideline-based	NMIBC (CIS; high-risk Ta/T1)	FDA prescribing information; guideline framework	[89,90]

## Data Availability

No new data were created or analyzed in this review. Data sharing is not applicable.

## Abbreviations

The following abbreviations are used in this manuscript:

AC-NPs	Antigen-capturing nanoparticles
AhR	Aryl hydrocarbon receptor
APC	Antigen-presenting cell
ASCO	American Society of Clinical Oncology
BCG	*Bacillus Calmette–Guérin*
CARD9	Caspase recruitment domain-containing protein 9
CD	Cluster of differentiation
cDC1	Conventional type 1 dendritic cell
CIS	Carcinoma in situ
CMC	Chemistry, manufacturing, and controls
CR3	Complement receptor 3
CRISPR	Clustered regularly interspaced short palindromic repeats
CTL	Cytotoxic T lymphocyte
DAMPs	Damage-associated molecular patterns
DC	Dendritic cell
DC-SIGN	Dendritic cell-specific intercellular adhesion molecule-3-grabbing non-integrin
EVs	Extracellular vesicles
FDA	United States Food and Drug Administration
GM-CSF	Granulocyte–macrophage colony-stimulating factor
GPCR	G protein-coupled receptor
hATTR	Hereditary transthyretin-mediated amyloidosis
HIF-1α	Hypoxia-inducible factor 1-alpha
ICI	Immune checkpoint inhibitor
IL	Interleukin
IL-1β	Interleukin-1 beta
IL-6	Interleukin-6
IL-7	Interleukin-7
IL-10	Interleukin-10
IL-12	Interleukin-12
IL-15	Interleukin-15
IL-21	Interleukin-21
iNeST	Individualized neoantigen-specific immunotherapy
IV	Intravenous
KDM5	Lysine-specific demethylase 5
LN	Lymph node
LNP	Lipid nanoparticle
LPS	Lipopolysaccharide
MDSCs	Myeloid-derived suppressor cells
MHC	Major histocompatibility complex
mRNA	Messenger RNA
mTOR	Mechanistic target of rapamycin
NF-κB	Nuclear factor kappa-light-chain-enhancer of activated B cells
NMIBC	Non-muscle-invasive bladder cancer
PAMPs	Pathogen-associated molecular patterns
PDI	Polydispersity index
PEG	Polyethylene glycol
PRR	Pattern recognition receptor
PXR	Pregnane X receptor
QC	Quality control
RNA	Ribonucleic acid
ROS	Reactive oxygen species
SCFAs	Short-chain fatty acids
siRNA	Small interfering RNA
STING	Stimulator of interferon genes
Syk	Spleen tyrosine kinase
TAMs	Tumor-associated macrophages
Th	T helper cell
TLR	Toll-like receptor
TME	Tumor microenvironment
TNF	Tumor necrosis factor
Treg	Regulatory T cell
4-1BBL	4-1BB ligand
